# Reliable imputation of spatial transcriptomes with uncertainty estimation and spatial regularization

**DOI:** 10.1016/j.patter.2024.101021

**Published:** 2024-07-09

**Authors:** Chen Qiao, Yuanhua Huang

**Affiliations:** 1 School of Biomedical Sciences, University of Hong Kong, Pokfulam, Hong Kong SAR, China; 2 Department of Statistics and Actuarial Science, University of Hong Kong, Pokfulam, Hong Kong SAR, China; 3 Center for Translational Stem Cell Biology, Hong Kong Science and Technology Park, Hong Kong SAR, China

**Keywords:** spatial transcriptomics, gene imputation, uncertainty estimation, spatial regularization

## Abstract

Imputation of missing features in spatial transcriptomics is urgently needed due to technological limitations. However, most existing computational methods suffer from moderate accuracy and cannot estimate the reliability of the imputation. To fill this research gap, we introduce a computational model, TransImpute, that imputes the missing feature modality in spatial transcriptomics by mapping it from single-cell reference data. We derive a set of attributes that can accurately predict imputation uncertainty, enabling us to select reliably imputed genes. In addition, we introduce a spatial autocorrelation metric as a regularization to avoid overestimating spatial patterns. Multiple datasets from various platforms demonstrate that our approach significantly improves the reliability of downstream analyses in detecting spatial variable genes and interacting ligand-receptor pairs. Therefore, TransImpute offers a reliable approach to spatial analysis of missing features for both matched and unseen modalities, such as nascent RNAs.

## Highlights

• TransImpute predicts unmeasured genes or modalities in spatial omics from scRNA-seq
• TransImpute achieves high accuracy and efficiency as a linear low-rank translation
• TransImpute estimates uncertainty of imputation, prioritizing reliably imputed genes
• TransImpute preserves spatial autocorrelation, avoiding overestimating spatial patterns

## The bigger picture

Spatial transcriptomic technologies allow researchers to measure the expression of many genes at once within a field of cells or a tissue and then to use that information to resolve cell types, study cell-cell signaling, and explore tissue biology. These technologies have a number of applications to medical research. Current technologies suffer from multiple limitations, resulting in missing genes or high noise, particularly as researchers push for increased spatial resolution. This makes imputation—replacing missing data with estimated values—necessary for those poorly measured or unmeasured features. Multiple imputation methods have been introduced, but their performance varies from gene to gene. Newer methods that handle imputation uncertainty in a more rigorous manner could help researchers prioritize meaningful findings and reduce false positives.

## Teaser

For multiple mainstream spatial transcriptomics platforms, it is necessary to impute the unprobed genes, modalities, or even noisy measurements. Multiple computational methods have been proposed with moderate but saturating performance, partly due to a lack of relevant information for some prediction targets. Here, the authors developed TransImpute for this task, focusing on estimating the imputation reliability and introducing spatial regularization, hence allowing users to prioritize the more predictable genes for biological discovery.

## Introduction

A variety of biological processes are modulated through the spatial organization of cells, including how different cell types are distributed in a microenvironment and how cells communicate and perform a cooperative biological function. Prominent examples include localization of cell types in mouse organogenesis^1^ and human thymus development,^2^ as well as intercellular communications in squamous cell carcinoma^3^ and during intestinal development.^4^

In recent years, the rapid development of spatial transcriptomics (ST) technologies has made it more accessible for dissecting the spatial mixture of cells in a wide range of biomedical research. The main two streams of technologies are sequencing based and imaging based (via *in situ* hybridization or *in situ* sequencing).^5^ The former, in principle, can cover the whole transcriptome but has a limited resolution of cells (e.g., around 5–10 cells per spot), while the latter can have a cell-level resolution but is generally limited to probing dozens of pre-selected genes.^6^ Recently, breakthroughs on both platforms, e.g., seqFISH+^7^ and Stereo-seq,^8^ are addressing these limitations in different aspects. However, RNA capture efficiency is still far from perfect in sequencing-based methods, and laborious designing of candidate gene probes is required in imaging-based methods.

Therefore, computational methods for feature imputation are highly demanded in analyzing ST data, particularly by leveraging the rich single-cell RNA-sequencing (scRNA-seq) data as a reference, including imputing unseen genes in imaging-based data or imputing poorly covered genes in sequencing data. In general, modality integration methods can be applied for the task of missing feature imputation, e.g., Liger^9^ and Seurat v.3.^10^ Recently, multiple tailored methods have also been proposed to address this challenge with improved performance reported.

For example, SpaGE imputes missing ST data by averaging k-nearest neighbors (kNNs) from the reference scRNA-seq data after projecting both ST and scRNA-seq datasets into a common low-dimensional space spanned by adapted principal vectors.^11^ Similarly, kNN-based aggregation strategies are also applied in a joint representation space produced by either shared principal components^1^ or the latent encodings of an autoencoder.^12^ Tangram is another appealing method that directly learns a mapping matrix for cells from scRNA-seq to spots in ST by minimizing the cosine distances at both feature and sample levels between imputed and observed ST expressions.^13^

Moreover, in addition to certain molecular features, mapping meta-information of cells, e.g., cell type labels, is also a task that shares the same principles of feature imputation but is usually treated as a standalone task, e.g., in RCTD^14^ and Cell2location.^15^ Broadly speaking, recent studies focusing on more challenging scenarios of multi-omics mosaic integration (e.g., scMoMat^16^ and MIDAS^17^) can also be applied for imputing missing genes in spatial data, as they provide a unified approach to both cell-type deconvolution and ST imputation tasks,^18^ where generative models^16^^,^^18^ and variational autoencoders^17^ are the major modeling tools.

However, multiple challenges in ST imputation remain less addressed. First, there is no indicator available for assessing the imputation reliability: it is not clear how reliable one imputed gene could be for further biological discovery. Second, most feature imputation methods do not explicitly consider the spatial pattern strengths during imputation, often resulting in overestimating spatial smoothness. Third, given the rapidly increasing number of cells in ST data, computational efficiency is another demanding property.

To address these challenges, we introduce a generic framework, TransImpute (TransImp for short), to transform information from an scRNA-seq reference to the ST context, with two major innovations. First, it can provide uncertainty scores for imputation performance, hence allowing us to focus on genes with more confident imputation. Second, it introduces a regularizer for spatial pattern preservation, alleviating the overestimation of spatial autocorrelation. To demonstrate the effectiveness of our model, we focused on a few challenging tasks, including the prediction of the dominant proportion of missing features in image-based ST datasets. We also verified its high reliability in common downstream spatial analyses: detection of spatially variable genes and interacting ligand-receptor pairs. Finally, we briefly showcase that this method can also be applied to the prediction of unspliced RNAs, hence enabling trajectory analysis of cell differentiation in a physical space.

## Results

### TransImpute model for ST imputation and uncertainty inference

In the TransImpute model, we aim to learn a mapping (i.e., translation) function f(·) to translate the scRNA-seq reference to ST data. It is worth clarifying that we use “spot” for ST data and “cell” for single-cell (SC)-reference data, although a “spot” may mean different things at different ST platforms. In imaging-based ST data, spots are pixels that capture cells, while in flow-cell-based systems like Visium, a spot means a barcoded region of cells. For simplicity and consistency, hereafter we intentionally use “spot” for all ST data and “cell” for all SC-reference data.

**Figure 1:**
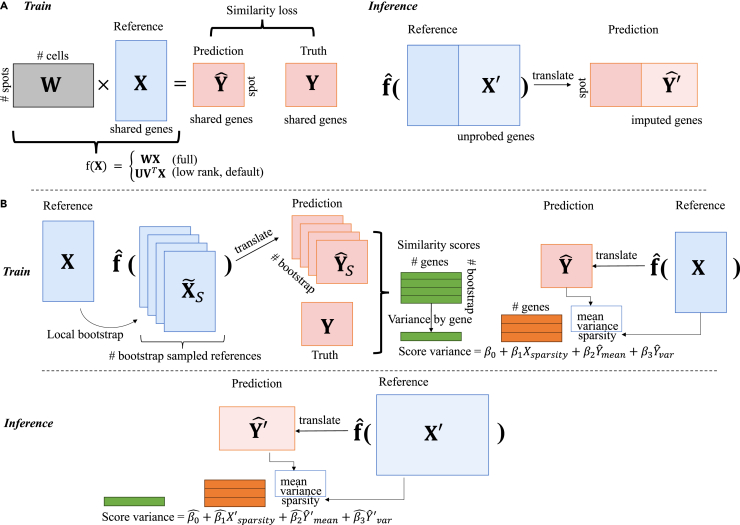
Illustration of the TransImpute (TransImp for short) computational framework (A) TransImp is broadly a low-rank linear mapping, serving translation from scRNA-seq to ST data. The mapping matrix W (or its low-rank factorization $UV^{T})$ will be achieved by using the overlapping genes between scRNA-seq and ST data. Once the mapping matrix is fitted, as denoted by $\hat{f}\left(\cdot \right)$, it can be used to perform the inference of the unprobed genes in ST data. (B) Quantification of the imputation uncertainty and how it can be predicted by a *post hoc* model. In the training stage, bootstrapping is performed by resampling SC cells locally within each cluster, creating multiple sampled references that are translated via fitted $\hat{f}\left(\cdot \right)$ to ST data. Each ${\hat{Y}}_{S}$ can be measured with a similarity score against the ground truth Y, from which a “score variance” over bootstrapped samples can be computed for each gene in the training set. A linear-regression model is then fitted based on three independent variables to predict the variance. $X_{sparsity}$ is the proportion of zero count in scRNA-seq data for a gene, while ${\hat{Y}}_{mean}$ and ${\hat{Y}}_{var}$ are the mean and variance of the imputed ST gene expression from the non-bootstrapped original SC reference X. At the inference stage, the linear model can predict the variance of imputed genes.

Related to the Tangram model,^13^ our overall translation framework is to learn a linear mapping matrix *W* from $N_{c}$ reference cells to $N_{s}$ ST spots (Figure 1A). One can also view it as a multivariate regression model (i.e., multiple outcome variables) by treating genes as samples and cells as feature dimensions (see the difference of this dual problem in Figure S1). Here, we further simplified the translation function to be a linear model without bias and introduced two modes: full and low rank (see details under “method”). To ensure computational efficiency and model robustness, we only use the low-rank mode for the SC reference (referred to as TransImpLR or simply TransImpute as default) and the full mode for the cell cluster reference (referred to as cluster mode or TransImpCls). One may see that the cluster mode (TransImpCls) is a special case of the low-rank model by pre-defining the cell loading matrix V as the cell-type identity matrix.

Then, the translation function is trained on the overlapped genes between reference and spatial datasets by minimizing the cosine similarity loss between the predicted and the observed spatial expression matrices at both gene and spot levels. Once the translation function $\hat{f}\left(\cdot \right)$ is learned, it can be applied to impute those genes that are unseen in the ST data but observed in the SC-reference data (Figure 1A, right). Of note, this framework can be easily extended for adding regularization terms into the loss function, for example, a spatial regularization term based on spatial autocorrelation statistics, Moran’s *I* (M.I.), which we will discuss under “method” and show how it encourages the mapping function to preserve spatial patterns in translation.

The translation function alone, however, lacks an indicator of prediction confidence on those missing genes. Therefore, we propose a framework to estimate the prediction uncertainty as illustrated in Figure 1B. First, we estimated the variance of imputation performance (score variance) on the training genes (where we have the true ST expression) as a *post hoc* step relying on a fitted translation function and the same training SC reference and ST datasets.

Specifically, with the SC-reference matrix, we sample with replacement in each Leiden cluster the exact same number of cells within this cluster. After obtaining $N_{sim}$ sampled SC-reference matrices, the already estimated function $\hat{f}\left(\cdot \right)$ can translate all of them into the ST domain, where $N_{sim}$ newly imputed ST data are created. Now, with the observed ST matrix (Truth), we can make $N_{sim}$ prediction-ground truth pairs and calculate the cosine similarity scores (CSSs) for each gene (cosine similarity by columns of the two cell-by-gene matrices). Consequently, for each gene, there accumulate $N_{sim}$ CSSs, and we can hence calculate the variance statistics to measure how uncertain the imputation for a gene is. We aim to predict this variance as the dependent variable in a linear model, which, after fitting on the training genes’ variances, can infer for unseen test genes their potential variances of imputation quality. The model consumes three features for each gene: sparsity of gene reads from the reference count matrix, denoted as $X_{sparsity}$, and mean and variance of the imputation prediction $\hat{Y}$, denoted as ${\hat{Y}}_{mean}$ and ${\hat{Y}}_{var}$, respectively. Last, with the estimated uncertainty prediction model parameterized as $\hat{\beta }$, a gene’s performance uncertainty can be inferred by feeding the corresponding features of the observed reference and imputed ST expressions. The predicted uncertainty can serve as a criterion for selecting imputed genes for downstream analysis, for which a threshold can be determined by finding potential knee points on the curve of the median CSSs over uncertainty thresholds on a holdout set or through cross-validation; example plots are provided as Figure S2.

Overall, TransImpute contributes to reference-based spatial feature imputation with important features, including quality score estimation and spatial regularization, which not only provide a selecting criterion for reliable imputation but also enable spatial-pattern-preserved imputation. Moreover, the flexible architecture makes TransImpute computationally lightweight and efficient in the low-rank configuration and easy to extend to more sophisticated non-linear scenarios or scale up to larger datasets. We summarize the properties of TransImpute in comparison with other methods in Table S1.

### TransImpute contributes to state-of-the-art imputation and its estimated uncertainty prioritizes unprobed ST genes for reliable analysis

**Figure 2:**
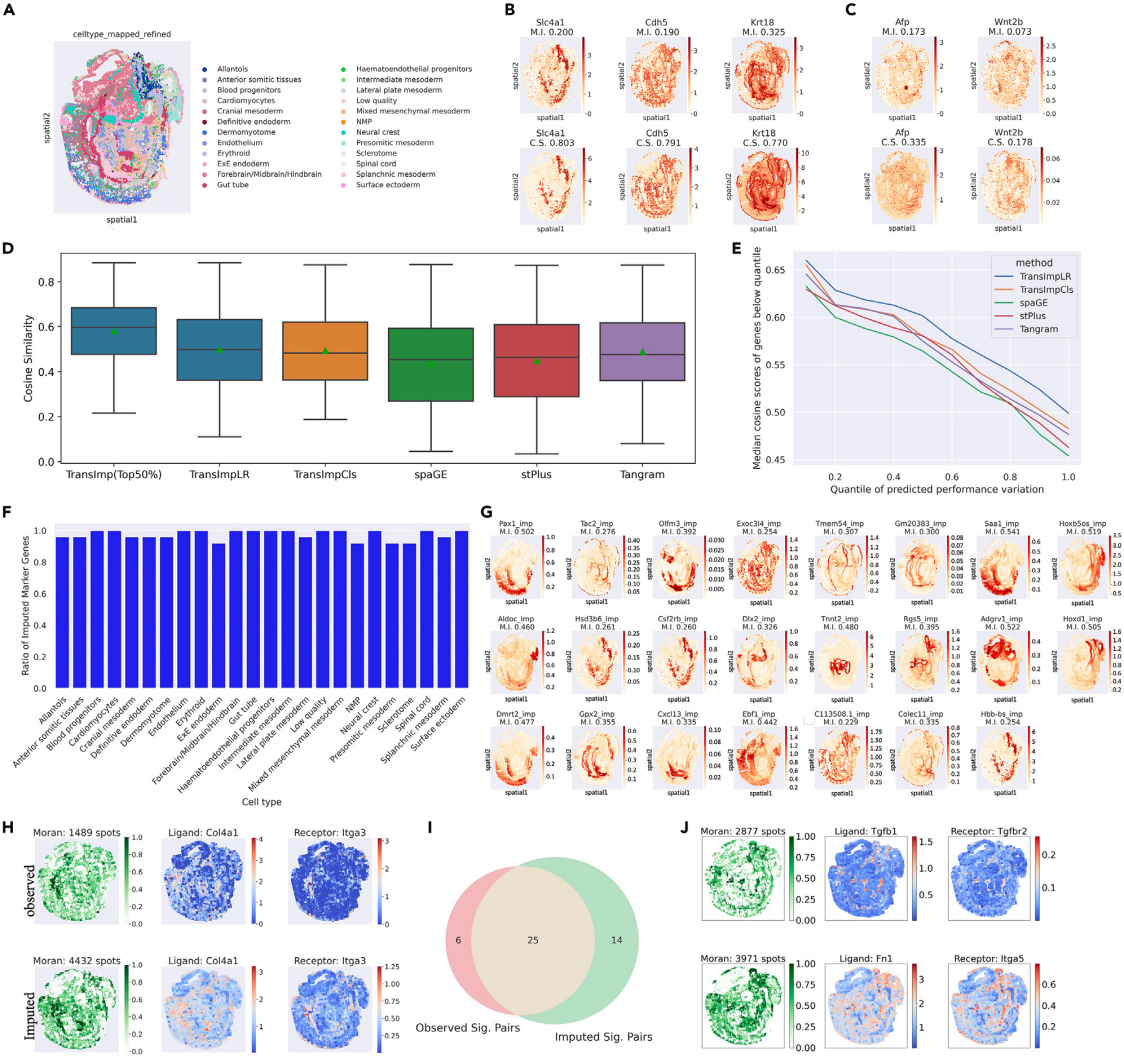
Evaluation results on seqFISH dataset (A) Observed cell-type distributions over spatial locations. (B) Example well-predicted genes: (top) observed, (bottom) imputed. M.I., Moran’s *I* statistics; C.S., cosine similarity score. (C) Example less-well-predicted genes: (top) observed, (bottom) imputed. (D) Boxplots of cosine similarity scores (CSSs) for all methods. The first box shows the statistics of a subset of genes, with predicted uncertainty below the median of all genes. (E) Line plot of CSSs aggregated in different quantile ranges. The x axis denotes the quantiles of predicted uncertainty, the y axis denotes the median CSS of genes below the corresponding uncertainty quantile. (F) Bar plot for proportions of unprobed marker genes in each cell type. (G) The top 1 ranked marker gene for each cell type. The suffix “imp” indicates unprobed genes imputed from the single-cell reference. (H) Spatial pattern plots of an example spatial ligand-receptor interaction pair: (top) on observed data, (bottom) on imputed data. In each row, the leftmost plot shows the probabilities of spatial interaction over spots, while the remaining two show the expression patterns of the involved genes. (I) Venn diagram of significant ligand-receptor pairs in observed and imputed ST expression matrices. (J) Plots of two spatial ligand-receptor pairs (top and bottom) of unprobed genes.

We first applied TransImpute to a dataset generated with the seqFISH platform on mouse organogenesis, where 351 genes were probed in 57,536 spots,^1^ covering 24 major cell types and their distributions, as shown in Figure 2A. To assess the imputation performance, we conducted a 5-fold cross-validation on these 351 genes and merged all the test folds for evaluation. In Figures 2B and 2C, we show example genes that are well and poorly imputed, respectively. The well-imputed genes tend to better capture the ground-truth spatial patterns, while those poorly imputed genes with either spurious or weak ground-truth spatial patterns challenge the model in prediction. An overall performance comparison for different imputation methods is visualized in Figure 2D, where the CSS indicates that the proposed method achieved the best performance regarding imputation consistency with ground truth (median CSS = 0.499 and 0.483 for TransImpLR and TransImpCls, respectively), significantly outperforming the existing methods at the Bonferroni-corrected 0.05 significance level in paired statistical tests (see Table S2), when using the same train-test split. We noticed that stPlus and Tangram also work comparably well (median CSS = 0.463 and 0.477, respectively), while SpaGE is less accurate on average (median CSS = 0.454).

Despite state-of-the-art performance being achieved, the overall accuracy was still not perfect, partly due to discrepancies between the reference and the ST datasets and technical measurement noise. Therefore, we asked if our proposed performance uncertainty surrogate could help identify more confident genes (see “method”). After ranking genes by their performance uncertainty, we found that the median CSS can be substantially improved from 0.499 to 0.600 by focusing on the half of the gene set with lower uncertainty (Figure 2D). In more detail, by plotting the median CSS of the remaining genes over the uncertainty quantile thresholds ranging from 10%, 20%, …, to 100% in Figure 2E, a negative association trend is evidently presented: the median CSS of genes at lower uncertainty quantiles tends to be higher, and it is not only for our method but also for all other methods, suggesting that our proposed performance uncertainty is an effective indicator of imputation confidence.

Given the enhanced accuracy of our uncertainty-aware imputation, we further explored to what extent the imputed genes would facilitate the biological analysis, including the cell-type marker genes and spatial ligand-receptor interaction. When examining the marker genes from the pool of observed and imputed genes, we found that >90% of markers are imputed genes for all cell types (Figure 2F). The top-1-ranked marker genes for each cell type are shown in Figure 2G, which turn out to be unprobed genes for all cell types.

These results indicate that much richer information may be entailed in the unprobed genes and that imputation is one solution to the limitations of ST technologies.

We further investigated gene interactions over spatial locations and used SpatialDM^19^ for detecting significant spatial ligand-receptor interactions. We first ran the test on all probed genes, and Figure 2H shows a typical example pair that is significant in both observed and imputed expressions. The figure also shows that the spatial interaction patterns in imputed expressions are more widespread (4,432 vs. 1,489 significant spots with local communication). When assessing all ligand-receptor pairs, 57 pairs were covered in the 351 genes, among which 31 and 39 were identified as interacting pairs by using the observed and imputed expression, respectively (false discovery rate [FDR] < 0.1). In Figure 2I, the Venn diagram further indicates a big overlap of significant pairs between observed and imputed data, implying the accuracy of imputation. Moreover, when leveraging the power of imputation on all unprobed genes, more significant interactions can be discovered (45 pairs), such as the two example pairs shown in Figure 2J, where active interaction regions cover cell types such as allantois and neural crest. The results again indicate the potential values of unprobed genes for biological discovery that can be achieved via imputation.

Finally, to investigate how unprobed genes can affect downstream clustering performance, we experimented on the seqFISH ST dataset with imputed genes that were not included in the seqFISH ST set. To prevent picking up a candidate gene set that may bias toward certain cell types at the single-cell level, we selected for each single-cell cluster its top 30 scored marker genes (363 in total, of which 217 were unprobed) after obtaining the top 3,000 highly variable genes. Combining all markers with the 351 ST genes yields a new set of 568 genes. After imputation, we conducted agglomerative clustering with adjacency matrices calculated using the spatial coordinates at the ST level. The performance was measured in four clustering metrics, and their averages are shown in Table S3. The result demonstrates several benefits of gene imputation with TransImp. First, clustering on the imputed marker gene subset achieved an 11.3% higher performance (mean score 0.3378) than clustering on the raw seqFISH ST genes (mean score 0.3036), indicating the value of unprobed genes for downstream analysis. Second, by comparing clustering results on subsets of confident genes, we observed that removing less confident imputations did benefit downstream clustering, with the best performance (15.8% higher than raw seqFISH) achieved when the top 300 confident genes were retained (mean score 0.3515). However, if too many marker genes were filtered out, the clustering would be affected, since some clusters may have lost important markers. This observation may explain the trend of decreasing performance from the top 200 confident genes onward, where smaller subsets of genes were used. In this scenario, in gene selection, we should also consider balancing the number of markers remaining in each cell cluster.

### TransImpute is efficient and robust across datasets from multiple platforms

**Figure 3:**
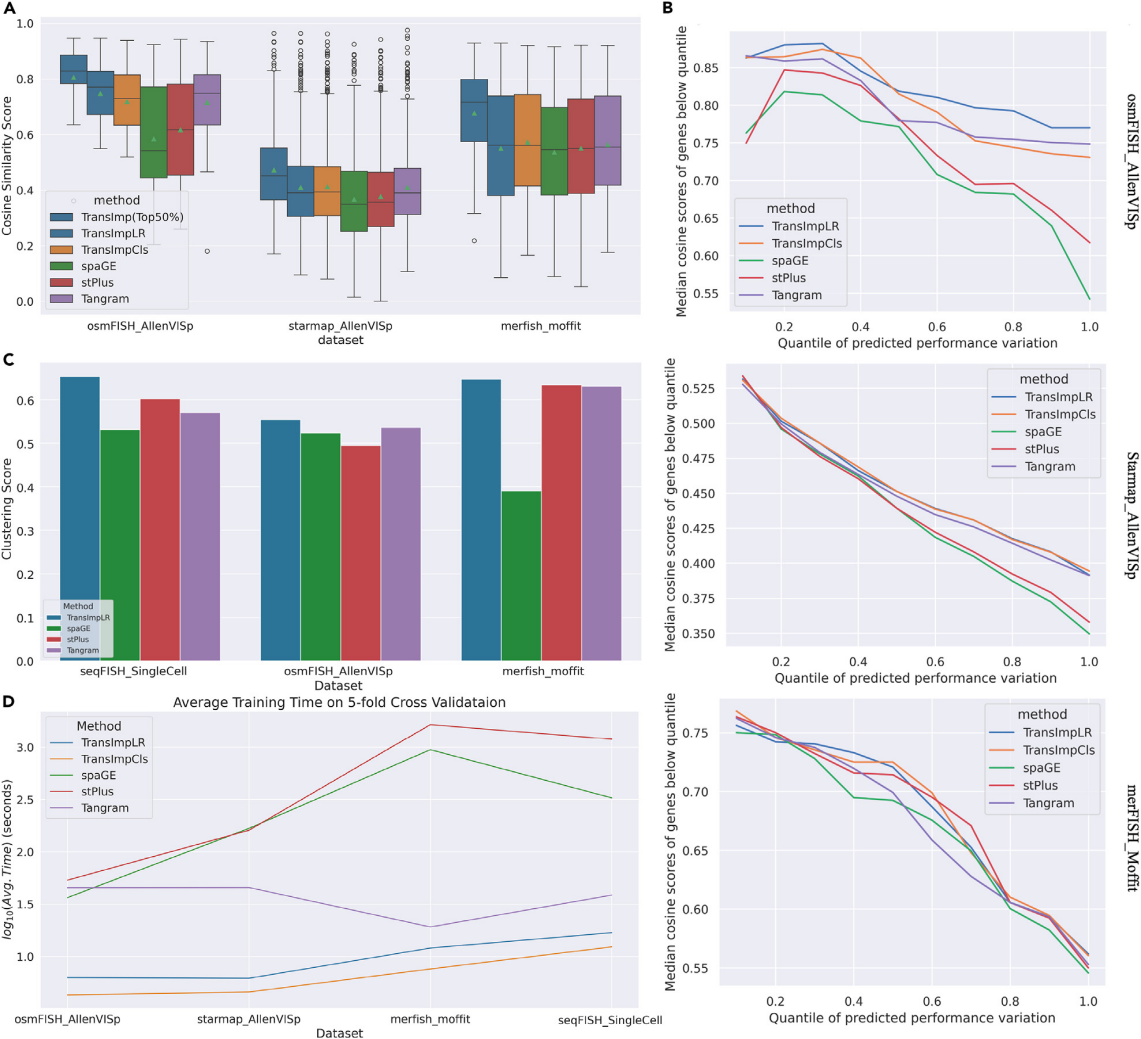
Evaluation of imputation methods (A) Cosine similarity scores (CSSs) for OsmFISH, STARmap, and MERFISH spatial transcriptomic datasets. (B) Line plots of imputation performances in different uncertainty quantile ranges, where the x axis denotes the quantiles of predicted uncertainty and the y axis denotes the median cosine similarity score of genes below the corresponding uncertainty quantile. (C) Averaged clustering scores from multiple metrics (“method”). (D) Averaged computational runtime of different methods.

Next, we conducted evaluation experiments on three more ST datasets generated using different technologies. In Figure 3A, we summarize the CSSs of gene profiles across datasets and methods in boxplots. It is evident that our methods TransImpLR/TransImpCls consistently achieved the best performance compared to those state-of-the-art methods in CSSs. Moreover, the predicted uncertainty did well in identifying reliably imputed genes, as demonstrated in the line plots of Figure 3B, where genes with more certain performance at the lower quantiles tend to achieve higher CSSs. The negative associations shown in the sub-figures are aligned with Figure 2E, indicating that the effective indicator is also generalizable to different platforms. When selecting the 50% of genes with lower predicted uncertainty, we can find in Figure 3A (the first boxes) that they have much higher median CSSs than the CSS of all the genes (e.g., 0.718 vs. 0.562 on MERFISH data). In Tables S2 and S4, we show the results of significance tests on comparing TransImpLR (complete gene set/top 50% certain genes) with other methods. The test results show that TransImp is the only method that can either significantly outperform other methods or remain comparable (no statistically significant differences). Moreover, TransImp, the only method that can provide quality scores for its predictions, successfully selected high-quality genes that significantly outperformed the full gene sets imputed by all other methods (Table S4), indicating its robustness.

Moreover, downstream clustering analysis was conducted on the imputed genes from the 5-fold test sets. To involve spatial information in the clustering, we adopt agglomerative clustering with adjacency matrices calculated using the spatial coordinates. We compare the clusters of imputed vs. true expressions after applying the same agglomerative clustering procedure. The averaged clustering metrics (covering multiple scores, e.g., adjusted rand index; see “method”) are visualized in Figure 3C, demonstrating that our method TransImpLR consistently achieved the best performance across all three datasets, particularly with clear gain on the seqFISH dataset (0.653 vs. 0.602 as the second best).

Finally, we recorded the training runtime for each method. As shown in Figure 3D, benefiting from graphics processing unit (GPU) acceleration, Tangram and our proposed methods are much more efficient than stPlus and SpaGE, particularly on larger datasets such as MERFISH and seqFISH. Between Tangram and our method, we found that both TransImpLR and TransImpCls can still achieve 37.1%–90.5% running time reduction, probably thanks to the low-rank setting.

### Spatial regularizer preserves spatial autocorrelation, reinforcing the downstream signal detection

**Figure 4:**
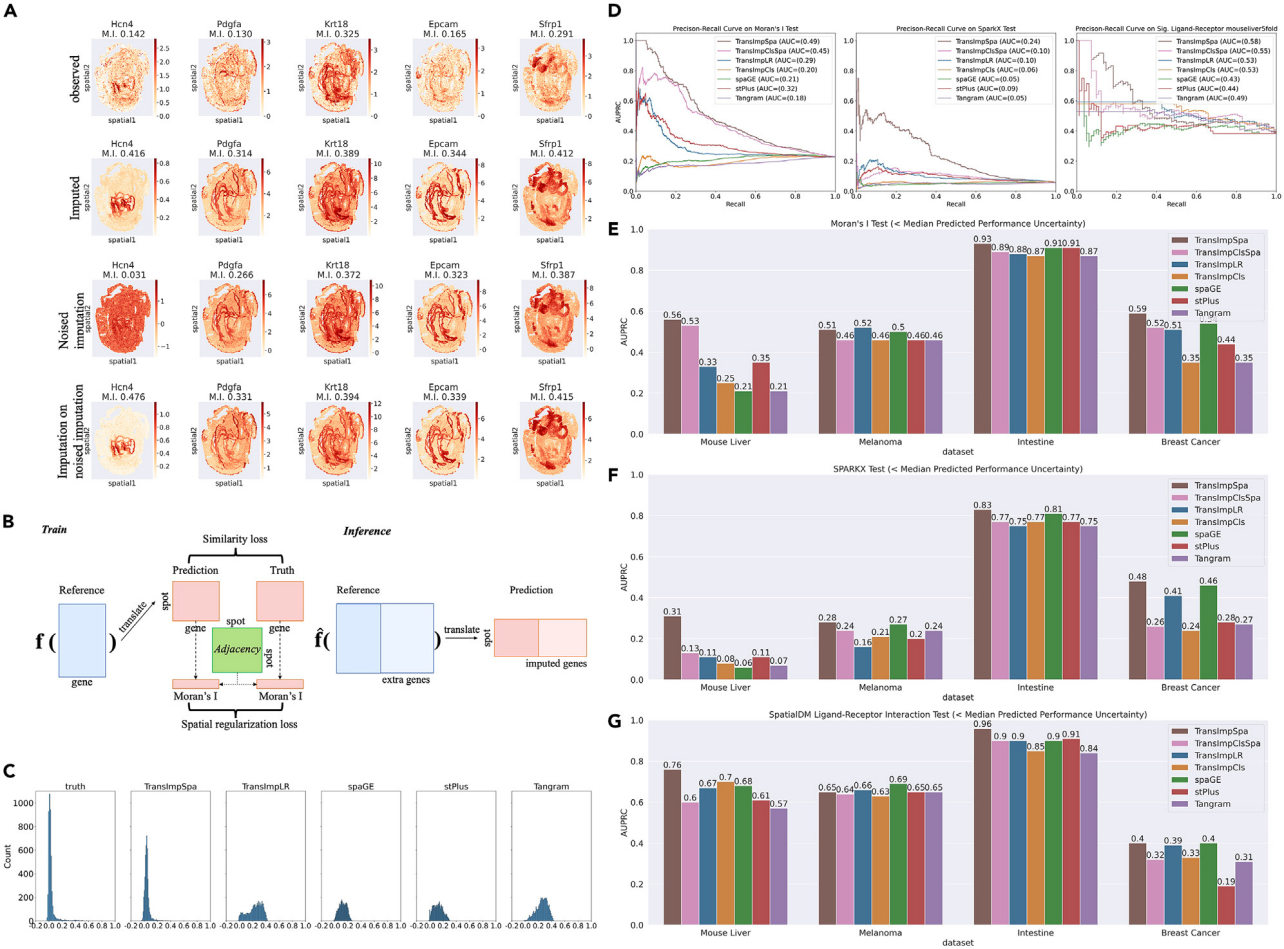
Spatially regularized imputation and experimental results (A) Example genes from the simulation experiment for demonstrating the denoising property of imputation. Top row: observed gene expression. Second row: imputed gene expressions that tend to have stronger Moran’s *I*-detected patterns. Third row: white-noised version of imputed genes from the above row, with spatial patterns weakened. Bottom row: imputation results with the noised genes as training data; the spatial patterns are much higher than the noised observations. (B) The training and inference procedures of the spatially regularized imputation. (C) Histogram plots of Moran’s *I* on a mouse liver dataset, indicating that the spatial regularization enables the predictions to have more consistent Moran’s *I* patterns. (D) From left to right, precision-recall curves and their area under the curve (AUC) scores of Moran’s *I* spatially highly variable gene (SHVG) detection tests, of Spark-X SHVG detection tests, and of spatial ligand-receptor interaction (SLRI) detection tests on the mouse liver dataset. (E–G) Bar plots of area under precision-recall curve scores for Moran’s *I* SHVG tests, Spark-X SHVG tests, and SLRI tests. Shown are results derived from genes with predicted performance uncertainty below the median. Note: performances on the mouse liver ST in (D) were measured on all imputed genes, whereas in (E)–(G), we report the performances measured on genes with imputation uncertainty below the median. Improved performances in (E)–(G) compared to (D) are meant to demonstrate that the uncertainty estimation enabled by TransImpute can indeed select better-imputed genes for downstream analysis.

Although TransImpute and other methods allow the imputation of missing genes, in empirical analyses we constantly find a common interesting phenomenon: the spatial patterns of the imputed gene expressions tend to be overestimated and hence exhibit stronger Moran’s *I* statistics than observed. Taking TransImpLR on the seqFISH dataset as an example, the imputation methods increase the spatial autocorrelation Moran’s *I* index from observed to imputed expressions on the test set (Figure 4A, top two rows). Interestingly, this trend was also true if we treated the imputation as clean ground truth and added synthetic noise to make a new training set, where we found again that the imputed data achieved much higher Moran’s *I* statistics (Figure 4A, bottom two rows) than the noised observation.

Overestimating the spatial pattern can be a risk for downstream biological analyses, and an ideal model should retain spatial patterns as consistent as possible with the observation. To achieve this goal, we propose to add a spatial regularization to the training objective. As an analogy to the regularizer used in LASSO and the ridge regression model, we anticipate that a spatial-pattern-based regularizer may prevent the model from overestimating spatial patterns. The auxiliary training loss is illustrated in Figure 4B. As shown in the figure, in addition to the standard similarity loss, a spatial regularization loss based on Moran’s *I* is enrolled to make the *I*s of the observed and predicted expressions consistent. The strength of regularizing could be adjusted by tuning its weight (see “method”). After estimating $\hat{f}\left(\cdot \right)$, the standard inference process applies seamlessly as in Figure 1A. To validate, we applied it to a real-world dataset and found (see Figure 4C) that other methods without spatial regularization have distribution mass on much larger Moran’s *I*s than the observed ground truth (the overestimation phenomenon), while spatially regularized TransImp, i.e., TransImpSpa, has distributions closer to ground truth.

Next, we examine whether or to what extent spatial regularization can improve the accuracy of downstream analysis for biological pattern discovery. Specifically, we applied both spatially regularized and unregularized configurations of our method (with or without the “Spa” suffix) together with other methods to four Visium-based ST datasets. Thanks to Visium’s capacity to sequence almost the whole transcriptome, far more genes can be captured, and we hence can obtain enough positive and negative observations for assessing the detection of spatially highly variable gene (SHVG) and spatial ligand-receptor interaction. Similar to the analyses above, we used the results from observed gene expression as ground truth and examined the correctness of that from imputed expressions. On the mouse liver dataset, Figure 4D demonstrates the better spatial pattern preservation performance of TransImpSpa with the auxiliary regularization. Overall, the tasks are extremely challenging for all the methods, yet TransImpSpa continues to obtain the best results. Specifically, for spatially highly variable gene detection (Figure 4D, left and middle), TransImpSpa achieved the highest area under the precision-recall curve (AUPRC) with a testing metric by using either Moran’s *I* (AUPRC: 0.49 by ours vs. 0.32 by others) or Spark-X (AUPRC: 0.24 vs. 0.09), corroborating its effectiveness in retaining spatial patterns for individual genes. Moreover, for spatial ligand-receptor interaction detection using SpatialDM (Figure 4D, right), TransImpSpa also outperforms all the other methods by a large margin (AUPRC: 0.58 vs. 0.49), showing its good spatial-pattern-preserving performance even in the intergene interaction context.

In addition to the mouse liver datasets, we further examined the robustness of the spatial regularization on three human datasets: melanoma, intestine, and breast cancer. The overall AUPRC performance scores for all the methods are shown in Figures 4E–4G for Moran’s *I*, Spark-X, and ligand-receptor interaction tests, respectively. Of note, we used predicted uncertainty to filter out genes above median uncertainty, since Visium ST datasets are of very high dimension and low quality. It is noticeable from the bar charts that TransImpSpa robustly outperforms other methods without spatial regularization in finding spatially highly variable genes with either Moran’s *I* or Spark-X methods, and it also remains the robust high-performing method for detecting spatial ligand-receptor interactions.

### TransImpute facilitates spatial RNA velocity analysis by imputing unspliced and spliced RNAs in spots

Finally, as a generic translation framework, TransImpute may have the capability of translating the unseen feature modality in the ST data from the reference scRNA-seq. Here, we specifically assess how the unspliced RNA (and the spliced RNA) abundance can be translated from scRNA-seq data to ST data.

To explore, we trained the TransImpSpa framework on the chicken heart and mouse brain datasets with corresponding reference datasets retrieved from Abdelaal et al.^20^ On each dataset, the model was trained on the anchor genes (shared between scRNA-seq and ST) and then used to predict the unspliced and spliced expression matrices into the spatial space, followed by RNA velocity analysis with scVelo stochastic mode.^21^

**Figure 5:**
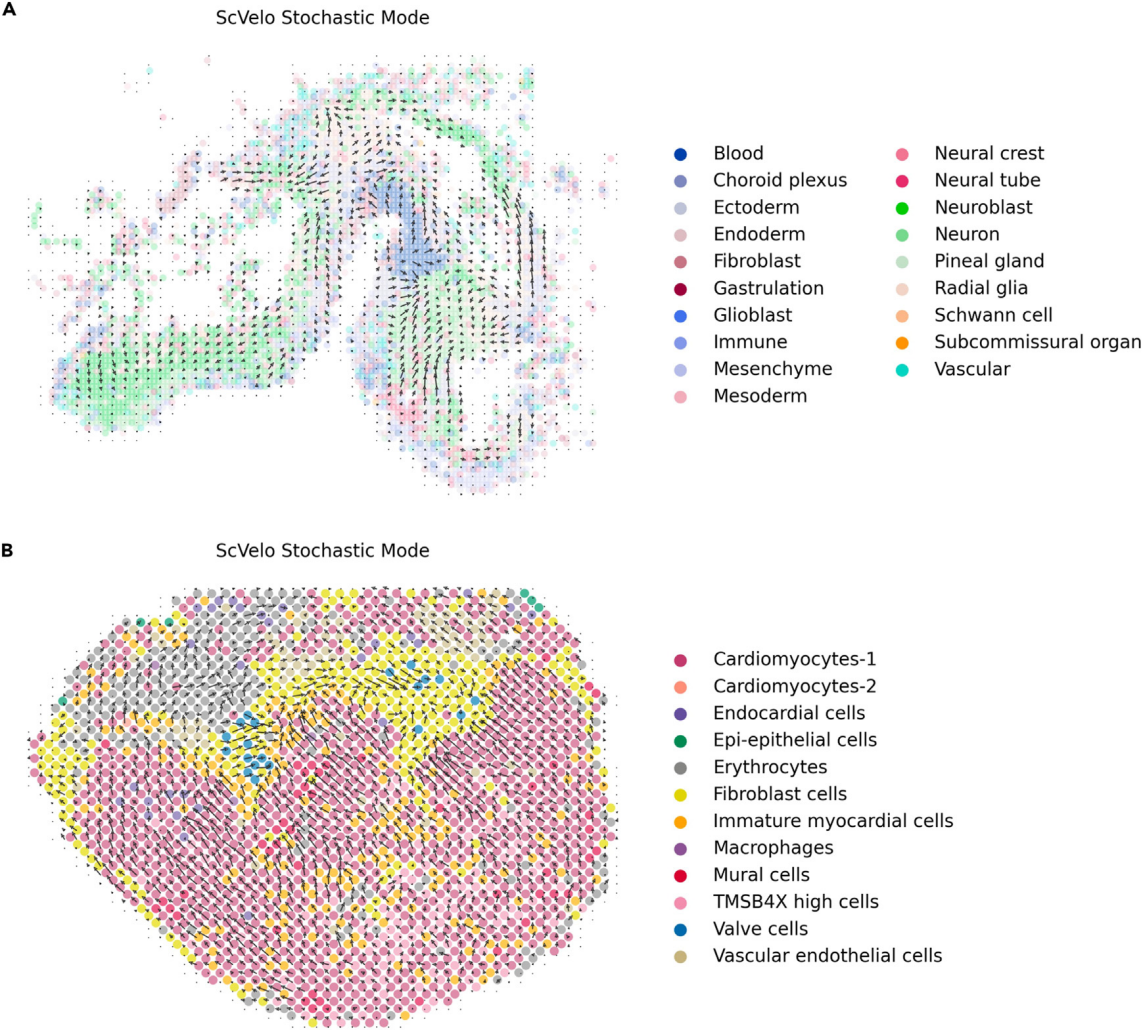
Spatial RNA velocity analysis based on imputed nascent and mature RNA counts Spot cell types are mapped from single-cell annotation. (A) Spot-level transition grid map on the mouse brain ST dataset. Sub-regions of the map show transitional directions from neuroblast to neuron captured at the single cell level. (B) Spot-level transition grid map on the chicken heart ST dataset. The overall trends of terminating at fibroblasts are consistent with prior work.

On the mouse brain dataset, we first performed the clustering on the scRNA-seq data and translated the cell type to the ST data (same practice as used in Tangram). As there is a lack of ground truth of the unspliced and spliced RNA abundance, we evaluated the performance by comparing the consistency of the predicted differentiation directional between ST and scRNA-seq. In Figure 5A, we found that the neuron differentiation from neuroblast cells is well captured in both scRNA-seq and ST data (Figures S3 and S4).

By performing a similar analysis on the chicken heart dataset, where the cell types were provided, we found that the overall transition trends terminating at fibroblasts are consistent with the pseudotime analysis for the epicardial lineage in Mantri et al.^22^ Taken together, these results demonstrate the potential of spatial RNA velocity analysis translating unseen modalities using the proposed framework.

## Discussion

To summarize, we introduce a framework for imputing missing features from reference scRNA-seq data. The predicted performance uncertainty helps identify reliable imputations. On various datasets from different platforms, we demonstrate that the proposed framework achieves state-of-the-art prediction accuracy, with predicted genes with lower uncertainty being more reliable and the spatial regularization preserving the spatial patterns of the imputed features. A comparison between TransImpute and a more recent method, SpatialScope,^18^ indicated that our method achieves comparable results (Tables S5 and S6). This aligns with our argument that the prediction of unprobed genes has become relatively saturated due to limited information available; therefore, more efforts should be made to prioritize predictable genes and avoid overfitting (of spatial patterns). In this light, the wide applicability of our method in common downstream analyses and computational efficiency may further accelerate the analysis of increasingly popular ST data.

On the other hand, it remains an open challenge to better structure imputation models with the location information available from ST. First of all, there are other model families to be explored for this task, and our low-rank mapping matrix has high flexibility to be adapted to them. As briefly mentioned, the full translation framework working with a cluster-aggregated gene signature matrix (TransImpCls) can be viewed as a special case of the low-rank framework with a cell-by-gene reference matrix, in that the low-dimensional matrix V is fixed to be a binary matrix of shape cell-by-cluster. Each row of the matrix is a one-hot vector turned on at the dimension corresponding to this cell’s cluster type. The low-rank setting hence offers an additional interface for injecting prior knowledge (e.g., cell types) into the translation function, either via explicit regularization on V and/or U or through a Bayesian manner. Moreover, beyond a linear setting for the low-rank framework, non-linearity may also be added to the translation function via, e.g., non-linear activation functions after the dot product with V and/or U, increasing model capacity for more complex mapping.

Second, as we demonstrated, local bootstrapping allows us to estimate performance uncertainty, which not only can be predicted by empirical features but also serves as an effective indicator of the reliability of the imputation. Low accuracy in the imputation for some genes may be inevitable for (almost) all computational methods, as the key information may be missed due to immature technology, making the selection of accurate genes a necessary step for reliable biological discovery. Our results have shown that selected genes of higher imputation quality can achieve more accurate imputation results. They also benefit downstream analysis with improved performance, such as in the detection of spatially highly variable genes and spatial ligand-receptor interaction pairs. In addition, they can contribute to increased performance in clustering/annotation of ST data. Conversely, without any measurement of imputation quality, all imputed features would be treated equally well, potentially affecting biological analyses due to accumulated imputation noise and leading to unexpected results. As an initial remedy, TransImpute provides uncertainty estimation along with its imputation results, so that more reliable analyses can be conducted by filtering out noisy imputations and retaining more confident predictions. Considering that the number of missing genes is often large, this prioritization of more accurate genes can further push computational methods for real applications, even though we are still at the beginning of assessing and predicting performance uncertainty. Furthermore, rigorous Bayesian methods may also be introduced to quantify imputation uncertainty by directly computing the posterior distribution of the latent variables V and U together with a dispersion term for each gene, broadly in the form of Bayesian matrix factorization. On the other hand, more sophisticated approximations may be needed for Bayesian computing to efficiently capture the uncertainty of the mapping.

Third, considering that the estimation of imputation uncertainty is maturing, one may consider further extending the downstream analysis by weighting the genes with their imputation uncertainty. In general, this type of weighted analysis will be task-specific, e.g., clustering and spatially variable gene detection, and can be generally difficult to implement. Nevertheless, a relevant but simpler idea is to set binary weights by including or not including the genes, and we showed how the selection may affect downstream analysis in cell clustering (Table S3).

Last, for the spatial regularization module, one may further re-evaluate spatial metrics that play an important role in quantifying spatial patterns of gene expressions, based on which the discrepancies between prediction and truth can be measured. It should be noted that the properties of a spatial metric affect downstream analysis. In TransImpSpa, we leveraged global Moran’s *I* as a proxy for quantifying spatial patterns. However, studies have argued for more powerful metrics such as SpatialDE^23^ and Spark-X^24^ for mining spatial patterns. In addition, applying local spatial metrics such as local Moran’s *I* and Geary’s *C* can be achieved by enabling spot-centered mini-batch optimization for translation functions, instead of fully batched training resulting from calculating global spatial metrics. This allows scaling the spatially regularized frameworks to even larger datasets.

## Experimental procedures

### Resource availability

#### Lead contact

Requests for further information and resources and reagents should be directed to and will be fulfilled by the lead contact, Dr. Yuanhua Huang at yuanhua@hku.hk.

#### Materials availability

This study did not generate new unique reagents.

#### Data and code availability

• Pre-processed and generated data in this study have been deposited at Zenodo under https://doi.org/10.5281/zenodo.7347655 ^25^ and are publicly available as of the date of publication.
• All original code has been deposited in the project TransImpute as part of an open-source python package, TranSpa (translation-based spatial transcriptomics analysis) and freely available at https://github.com/qiaochen/tranSpa. For reproducibility, all analysis notebooks are also available in this repository with links to the pre-processed datasets and is publicly available as of the date of publication.
• Any additional information required to reanalyze the data reported in this paper is available from the lead contact upon request.

### Method

As shown in Figure 1A, a standard translation framework translates an input reference gene profile or signature into the target gene profile on spots, where we treat genes rather than cells/spots as an input instance. The translation function entails the simplest configuration where it is linear without bias terms (assuming both datasets are normalized to the same scale), reducing to an alignment/mapping matrix as inferred by Tangram.^13^ Nevertheless, a translation function could be more flexible as being low rank or even non-linear. Moreover, we may add spatial regularizations in fitting translation functions, so that they could better preserve spatial patterns.

#### Translation function

A translation function f(·) takes as input a reference gene profile x and outputs the target spatial profile y: y=f(x). This work considers simple settings of being full or low-rank linear mapping:

$$f\left(x\right)=\left\{ \begin{array}{cc} \text{Wx} & \text{full}\\ U\cdot V^{T}x & \text{low rank (default),} \end{array}\right.$$

where non-negative matrix $W\in R^{N_{s}\times N_{c}}$, $N_{s}$ and $N_{c}$ are the numbers of spots and cells, respectively. In the low-rank setting, W is approximated by the matrix multiplication of two low-dimensional non-negative matrices, $U\in R^{N_{s}\times K}$ and $V\in R^{N_{c}\times K}$, where *K* is a hyperparameter specifying the dimensionality. We constrain V to be non-negative by taking the element-wise square of an unconstrained matrix $\tilde{V}$, denoted as $V:={\tilde{V}}^{2}$. U is constrained by taking the softmax of an unconstrained matrix $\tilde{U}$ over the *K* latent dimensions. The design motivation is to first implicitly generate latent cluster centers (in space $R^{K}$) and then construct spots by weighted combinations of these centers. In addition, we consider two modes of the input x: (1) cluster mode, aggregating the reference matrix by summing over clusters provided by, e.g., the Leiden method, and (2) cell mode, the whole gene profile vector. For efficiency of computation, we apply low-rank mapping for the cell mode (as default) and full mapping for the cluster mode (a special case of the low-rank cell mode). Generally, in low-rank approximation, the smaller *K* is (e.g., k = 4, 8, 16, 32), the more likely the mapping matrix would underfit, hence, yielding relatively low performance due to poor approximation to the mapping matrix (Figure S5). On the contrary, if *K* is configured to be large, it may instead overfit to noises in the training set and be affected in imputation performance for unseen genes. In the current study, *K* is set to be 256, an empirical good value for the majority of datasets in terms of both effectiveness and efficiency.

#### Translation loss

Shared genes between reference and ST datasets are used for supervised training of the translation function. We denote the output of the translation function for all the genes as matrix $\hat{Y}$, which is compared with the ground-truth matrix Y. The translation loss is computed based on the cosine similarity between the rows and the columns of the two matrices, capturing both the spot-wise and the gene-wise expression distributions:

$$l_{trans}=\frac{1}{N_{s}}\sum_{i=1}^{N_{s}}\left(1-\cos\left({\hat{y}}_{i,:},y_{i,:}\right)\right)+\frac{1}{N_{g}}\sum_{j=1}^{N_{g}}\left(1-\cos\left({\hat{y}}_{:,j},y_{:,j}\right)\right)\text{,}$$

where $y_{i,:}$ and $y_{:,j}$ index the *i*th row and *j*th column from matrix Y, respectively. $N_{g}$ is the total number of shared genes.

#### Spatial regularization loss

To explicitly encode spatial patterns into the training procedure, we adopt global Moran’s *I*,^26^ a well-studied spatial autocorrelation metric, as the quantitative measurement and compare *I* values on predicted and true expressions of each gene using mean squared error (MSE) loss:

$$l_{spa}=\frac{1}{N_{g}}\sum_{i=1}^{N_{g}}{\left({\hat{I}}_{i}-I_{i}\right)}^{2}\text{,}$$

where ${\hat{I}}_{i}$ and $I_{i}$ are Moran’s *I* computed on predicted and true expressions of gene *i*, respectively (see Figure 4B).

#### Uncertainty estimation

To estimate the reliability of gene imputation, we propose a *post hoc* uncertainty prediction model as illustrated in Figure 1B. This is a linear-regression model designed to predict the uncertainty of imputation performance for each imputed gene. The dependent variable is performance uncertainty (score variance in the figure). For training data, this uncertainty measurement can be derived with a local bootstrapping procedure. With the SC reference matrix, we sample with replacement in each Leiden cluster the exact same number of cells within this cluster. After obtaining $N_{sim}$ sampled SC reference matrices, the already estimated function $\hat{f}\left(\cdot \right)$ can translate all of them into the ST domain, where $N_{sim}$ newly imputed ST data are created. Now, with the observed ST matrix (truth), we can make $N_{sim}$ prediction-ground truth pairs and calculate the CSSs for each gene (cosine similarity by columns of the two cell-by-gene matrices). Consequently, for each gene there accumulate $N_{sim}$ CSSs, and we can hence calculate the variance statistics to measure how uncertain the imputation for a gene is. We aim to predict this variance as the dependent variable in a linear model, which, after fitting on the training genes’ variances, can infer for unseen test genes their potential variances of imputation quality. The model consumes three features: sparsity of gene reads from the reference count matrix, denoted as $X_{sparsity}$, and mean and variance of the imputation prediction $\hat{Y}$ from the original SC reference, denoted as ${\hat{Y}}_{mean}$ and ${\hat{Y}}_{var}$, respectively. With these training data, the following linear-regression model can be trained:

(Equation 1) $$\text{Uncertainty}=\beta_{0}+\beta_{1}X_{sparsity}+\beta_{2}{\hat{Y}}_{mean}+\beta_{3}{\hat{Y}}_{var}\text{.}$$

With the trained model $\hat{\beta }$, a gene’s performance uncertainty can be inferred by feeding the corresponding three features into the model. We would expect those genes with smaller uncertainty to be more reliable, by assuming that the local resampling of the original SC reference matrix should affect reliably imputed genes less, since the local context should be more homogeneous for well-predicted genes.

#### Model configuration and training

Four settings of the proposed framework are studied and evaluated on different datasets. As shown in Table 1, configurations denoted as TransImpClsSpa and TransImpCls are cluster-based full mapping frameworks with and without spatial regularization, respectively. Likewise, TransImpSpa and TransImpLR are cell-based low-rank settings of the translation framework with and without spatial regularization.

**Table 1:** Model configurations

	Without Spa.Reg	With Spa.Reg
Low rank (cell mode)	TransImpLR	TransImpSpa
Full (cluster mode)	TransImpCls	TransImpClsSpa

For configurations with spatial regularization, a hyperparameter λ is used to balance the spatial regularization strength in the total loss:

$$l_{total}=l_{trans}+\lambda \cdot l_{spa}\text{.}$$

Only the translation loss $l_{trans}$ is used for configurations without spatial regularization, which can also be viewed as a special case of the total loss when λ=0. In addition, one may further customize the hyperparameter λ for the spatial regularization weight. Overall, we found that the default value 1.0 achieves a good balance between controlling overestimation of spatial pattern and preserving imputation accuracy, as shown in Figures S6 and S7.

All the models are implemented using pytorch 2.0^27^ and trained with the AdamW optimizer^28^ on a GPU.

#### Datasets and configuration

Two categories of ST datasets are used for evaluating the imputation performance of the proposed methods, as shown in Table 2. The top four rows summarize the imaging-based ST datasets. We obtained the pre-processed STARmap, MERFISH, and OsmFISH, as well as the corresponding references AllenVISp and Moffit from Abdelaal et al.,^11^ while the seqFISH dataset with its SC reference was obtained from Lohoff et al.^1^ The bottom four rows summarize the information of the Visium-based ST datasets. The pre-processed mouse liver ST c1 sample and its reference are from Vandenbon et al.^29^ The breast cancer ST sample 1142243F and SC reference are from Wu et al.^30^ and Gambardella et al.,^31^ respectively. We obtained the pre-processed human melanoma^32^ ST dataset and its reference as well as the intestine^33^ ST A1 sample from the SpatialDM authors,^19^ and the intestine reference was obtained from Wang et al.^34^

**Table 2:** Dataset information

Dataset	No. of spots	No. of cells	No. of Spa.genes	No. of Ref.genes
seqFISH_Single Cell	57,536	32,844	351	29,452
osmFISH_AllenVISp	3,405	14,249	33	34,617
starmap_AllenVISp	1,549	14,249	1,020	34,617
Merfish_Moffit	64,373	31,299	155	18,646
Visium mouse liver	2,110	4,759	16,225	19,355
Visium human intestine	2,649	14,537	33,538	19,525
Visium human breast cancer	4,784	35,276	28,402	33,745
Visium human melanoma	293	4,645	5,779	21,118

The spatial adjacency matrices of all the ST datasets were calculated using the function *squidpy.gr.spatial_neighbors()* from the python package Squidpy.^35^ Briefly, a spatial adjacency matrix is the kNN graph on tissue space (physical distance), with distances converted to adjacency weights between each neighbor pair. The spatial adjacency matrices were used for computing Moran’s *I* indices and for spatial agglomerative clustering.

For all the datasets, we train all the models with 2,000 epochs, a learning rate of 0.01, and a weight decay of 0.01 and set the latent dimension for low-rank modes to be 256. There are only two exceptional TransImpSpa models on Visium datasets; each requires one differently set hyperparameter: the latent dimension to be 128 on the melanoma dataset to make it further smaller than the number of spots, 293, and the elements of V clipped to be within 0.5 for the intestine dataset to prevent overfitting.

For RNA velocity analysis, the pre-processed versions of the two ST datasets with the corresponding SC references, Day 14 Chicken Heart^22^ and Developing Mouse Brain Atlas,^36^ were obtained from Abdelaal et al.^20^

#### Evaluation of imputation

We compare our method and previous methods, including stPlus,^12^ SpaGE,^11^ and Tangram,^13^ on 5-fold cross-validation results over different ST datasets. To measure the similarity between predicted and true gene profiles, the CSS is calculated for each gene and aggregated by the median (Figures 2D and 3A) within each dataset. In a transposed view, we also provide cell-level CSSs in Figures S8–S11.

#### Evaluation on spatially highly variable gene detection

To further evaluate the imputation methods, we assess the downstream task of detecting spatially highly variable genes from the imputed expression matrices. This evaluation can only be conducted on Visium-based ST datasets, due to their almost whole-genome-wide sequencing capacity that can capture enough positive and negative genes for analysis. We adopted both the classical Moran’s *I* test^26^ and the more recent non-parametric Spark-X test^24^ and set the significance level to FDR < 0.01 for both methods. Viewing significant and non-significant results as binary classification, we may draw precision-recall curves (PRC) (in Figures 4D, S12, and S13) and summarize the performances of different methods as the area under the curve (AUC) (in Figures 4E and 4F), which is a better metric than the area under the receiver-operating characteristic curve in scenarios of label imbalance.

#### Evaluation on spatial ligand-receptor pair detection

Beyond spatial patterns of individual genes, we evaluate methods in a more challenging task that tries to identify spatially interactive ligand-receptor pairs. The recently developed method SpatialDM^19^ leverages a bivariant Moran’s statistic to detect spatial co-expression patterns of ligand and receptor pairs and is used as the assessment tool in our evaluation. We set the significance level of FDR to 0.01, and after running the test for the ground truth and all the imputed expression matrices, we could also plot PRC and calculate the summary AUC for model comparison, as shown in Figures 4D, 4G, and S14.

#### seqFISH unprobed gene analysis

The SC reference dataset has 29,452 genes, from which we selected the top 1,000 highly variable genes and uniformly sampled 1,000 genes. The intersected genes, excluding those in the 351 probed seqFISH genes, amount to 1,754, which constitutes the final set of unprobed genes imputed from the SC reference. We combined probed (observed) and unprobed (imputed) ST genes into an extended seqFISH dataset and ran Wilcoxon-based marker gene detection and SpatialDM implemented spatial ligand-receptor interaction detection tests.

#### Spatial clustering evaluation

We further evaluated our methods on downstream clustering analysis. The analysis was conducted on OsmFISH, MERFISH, and seqFISH, where the annotation of cell types is available. Agglomerative clustering structured by a spatial adjacency matrix was applied to each dataset, including both the true and the predicted expression matrices. We then compared the clustering results of predicted and true expressions with averaged clustering indices of adjusted Rand score (ARS), adjusted mutual information score (AMIS), homogeneity score (HOMO), and normalized mutual information score (NMI) (in Figure 3C).

#### Spatial RNA velocity exploration

To explore the potential application of the proposed method on spatial RNA velocity analysis, we fit TransImpLR on reference datasets and translated its unspliced and spliced mRNA count matrices into the spatial space, where RNA velocity was inferred using scVelo.^21^

## Supplemental information

Document S1. Figures S1–S14 and Tables S1–S6

Document S2. Article plus supplemental information
