# Supplementary material for: Reliable imputation of spatial transcriptomes with uncertainty estimation and spatial regularization
Source: Patterns (N Y). 2024 Jul 9;5(8):101021. doi: 10.1016/j.patter.2024.101021 (PMC11368697; doi:10.1016/j.patter.2024.101021)
Supplement: Document S1. Figures S1–S14 and Tables S1–S6 [file mmc1.pdf]

Patterns, Volume *5*

Supplemental information

Reliable imputation of spatial transcriptomes with uncertainty estimation and spatial regularization

Chen Qiao and Yuanhua Huang

Supplementary Figures for "Reliable imputation of spatial transcriptomes with uncertainty estimation and spatial regularization"

Chen Qiao and Yuanhua Huang

Method	Properties<br>Explicit/Implicit mapping matrix	Quality score for imputation	Spatial regularization	Low-Rank approximating	Nonlinear extension	Mode for scaling up to larger dataset
TransImp	Y(E)	Y	Y	Y(E)	Y	Y
Tangram	Y(E)	-	-	-	-	-
SpaGE	Y(I)	-	-	Y(I)	-	-
stPlus	Y(I)	-	-	Y(I)	-	-

Table S1: The properties of TransImp in comparison to other methods.
Note: (I) indicates implicit estimation of a full or low-rank mapping matrix. SpaGE and stPlus try to project both ST and SC datapoints into a common latent vector space, where similarity can be computed and hence the mapping between spots and cells. However, there is not an explicitly estimated mapping matrix as in Tangram or TransImp, where we indicated with (E).

Dataset	compairison	tstats	ttpval	wstats	wcpval
SeqFISH	TransImpLR-Tangram	5.650475	3.319418e-08	16332.0	1.986883e-14
	TransImpLR-spaGE	23.955782	9.151020e-76	694.0	9.949286e-57
	TransImpLR-stPlus	18.957011	1.217357e-55	3263.0	8.881602e-48
osmFISH	TransImpLR-Tangram	2.599833	1.399599e-02	108.0	1.472702e-03
	TransImpLR-spaGE	7.666170	9.749080e-09	0.0	2.328306e-10
	TransImpLR-stPlus	4.979864	2.106917e-05	56.0	1.435960e-05
starmap	TransImpLR-Tangram	0.171549	8.638268e-01	243089.0	6.452078e-01
	TransImpLR-spaGE	25.766850	1.592237e-112	33592.0	3.825023e-123
	TransImpLR-stPlus	21.438314	4.284522e-84	48899.0	2.104539e-106
MERFISH	TransImpLR-Tangram	-2.067836	0.040350	5531.0	0.512577
	TransImpLR-spaGE	1.968816	0.050793	4735.0	0.035313
	TransImpLR-stPlus	-0.049533	0.960559	5607.0	0.605577

Table S2: Significance test of the imputation performance between TransImpLR and other methods across different datasets.
Note: Methods are compared on genes' cosine metric scores. TransImpLR is the only method that either significantly outperformed all other methods or remains comparable (no statistical significant differences) even with a conservative p value correction method like Bonferroni correction ($p_{adj} = 0.05/3 = 0.0167$).
Abbreviations: tstats: t statistics of paired t test; ttpval: p values of paired t test; wstats: statistics of Wilcoxon signed-rank test; wcpval: p values of Wilcoxon signed-rank test

	Mean	AMIS	ARS	HOMO	NMI
SeqFISH_Raw (351) vs Ground.Annotation	0.3036	0.3394	0.1839	0.3507	0.3405
SCImputedMarkers (363) vs Ground.Annotation	0.3378	0.3459	0.3290	0.3294	0.3470
SCImputedAll (568) vs Ground.Annotation	0.3309	0.3363	0.3249	0.3250	0.3375
SCImputedTopConfident (500) vs Ground.Annotation	0.3397	0.3453	0.3337	0.3333	0.3464
SCImputedTopConfident (400) vs Ground.Annotation	0.3444	0.3474	0.3463	0.3353	0.3486
SCImputedTopConfident (300) vs Ground.Annotation	0.3515	0.3531	0.3535	0.3454	0.3542
SCImputedTopConfident (200) vs Ground.Annotation	0.3410	0.3434	0.3420	0.3341	0.3445
SCImputedTopConfident (100) vs Ground.Annotation	0.3185	0.3267	0.3115	0.3078	0.3279
SCImputedTopConfident (50) vs Ground.Annotation	0.2830	0.3063	0.2481	0.2698	0.3076

Table S3: Performances of Agglomerative clustering with spatial adjacency matrices on SeqFISH raw and different subsets of imputed genes.
Note: Scores are computed agaist ground truth annotations provided in SeqFISH dataset. Numbers in brackets show the number of genes in the configuration.

Dataset	compairison	tstats	ttpval	ustats	utpval
SeqFISH	TransImp(Top50%)-Tangram	6.853256	2.034213e-11	41555.0	4.078745e-11
	TransImp(Top50%)-spaGE	8.882853	1.042065e-17	44000.0	5.974558e-16
	TransImp(Top50%)-stPlus	8.132002	3.073518e-15	42897.0	1.186602e-13
osmFISH	TransImp(Top50%)-Tangram	1.996808	0.051656	386.0	0.009587
	TransImp(Top50%)-spaGE	3.981930	0.000236	436.0	0.000256
	TransImp(Top50%)-stPlus	3.384295	0.001448	411.0	0.001788
starmap	TransImp(Top50%)-Tangram	8.279617	2.717230e-16	312368.0	7.464302e-17
	TransImp(Top50%)-spaGE	12.355726	1.859368e-33	343849.0	4.505877e-35
	TransImp(Top50%)-stPlus	11.430210	4.622882e-29	338493.0	1.754071e-31
MERFISH	TransImp(Top50%)-Tangram	4.424013	1.503819e-05	7822.0	2.115320e-05
	TransImp(Top50%)-spaGE	5.172276	5.075941e-07	8131.0	9.253096e-07
	TransImp(Top50%)-stPlus	4.562129	8.289238e-06	7859.0	1.486145e-05

Table S4: Significance test of TransImp(Top50%) imputation performance and other methods across different datasets.
Note: Cosine scores of top-50% quality genes imputed by TransImpLR are compared with other methods with independent t tests and Mann-Whitney U tests. Results show that imputation quality can vary across genes, and that TransImp successfully selected high quality genes, which significantly outperform the full gene sets imputed by other methods, demonstrating its robustness.
Abbreviations: tstats: t statistics of independent t test; ttpval: p values of independent t test; ustats: statistics of Mann–Whitney U test; utpval: p values of Mann–Whitney U test

Method	Cux2	Otof	Rorb	Rspo1	Sulf2	Fezf2	Osr1	Mean
spaGE	3.1787	0.3091	3.4663	0.5978	6.8946	1.2150	0.1443	2.2580
Tangram	3.0127	0.3115	3.3875	0.6104	6.4768	1.1755	0.1562	2.1615
SpatialScope	2.5946	0.3050	3.8804	0.5875	6.5991	1.1989	0.1574	2.1890
TransImp	2.9352	0.3110	3.2405	0.6215	6.4753	1.1630	0.2301	2.1395

Table S5: Imputation performance measured in Mean Absolute Error (MAE) on SpatialScope Benchmark

Method	Cux2	Otof	Rorb	Rspo1	Sulf2	Fezf2	Osr1	Mean
spaGE	0.8292	0.7430	0.6777	0.7180	0.8001	0.8187	0.3883	0.7107
Tangram	0.7610	0.6954	0.6474	0.6610	0.7861	0.7832	0.3768	0.6730
SpatialScope	0.7974	0.5643	0.4864	0.6052	0.6538	0.6278	0.1929	0.5611
TransImp	0.8056	0.7228	0.7444	0.7046	0.7962	0.7678	0.3815	0.7033

Table S6: Imputation performance measured in Cosine Similarity on SpatialScope Benchmark.

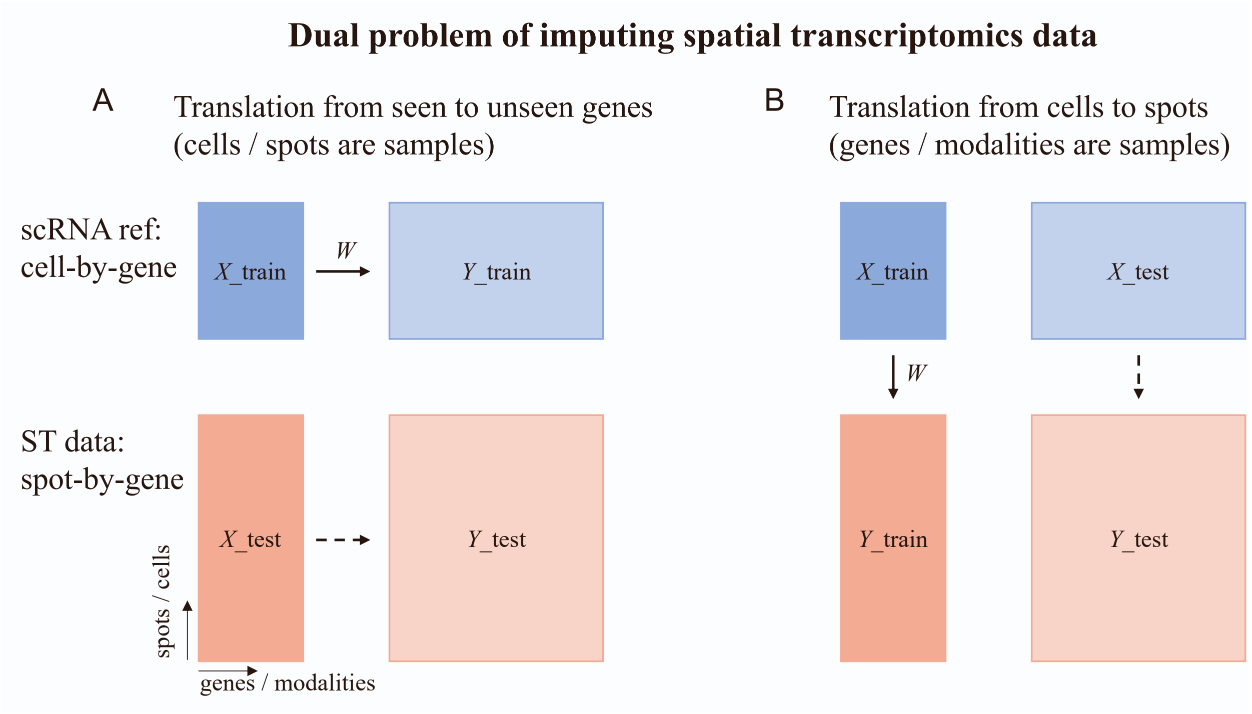

Figure S1: Dual problem of imputing spatial transcriptomics data. A. Translate observed genes to unobserved genes. B. Translate cells to spots, as used in our method.

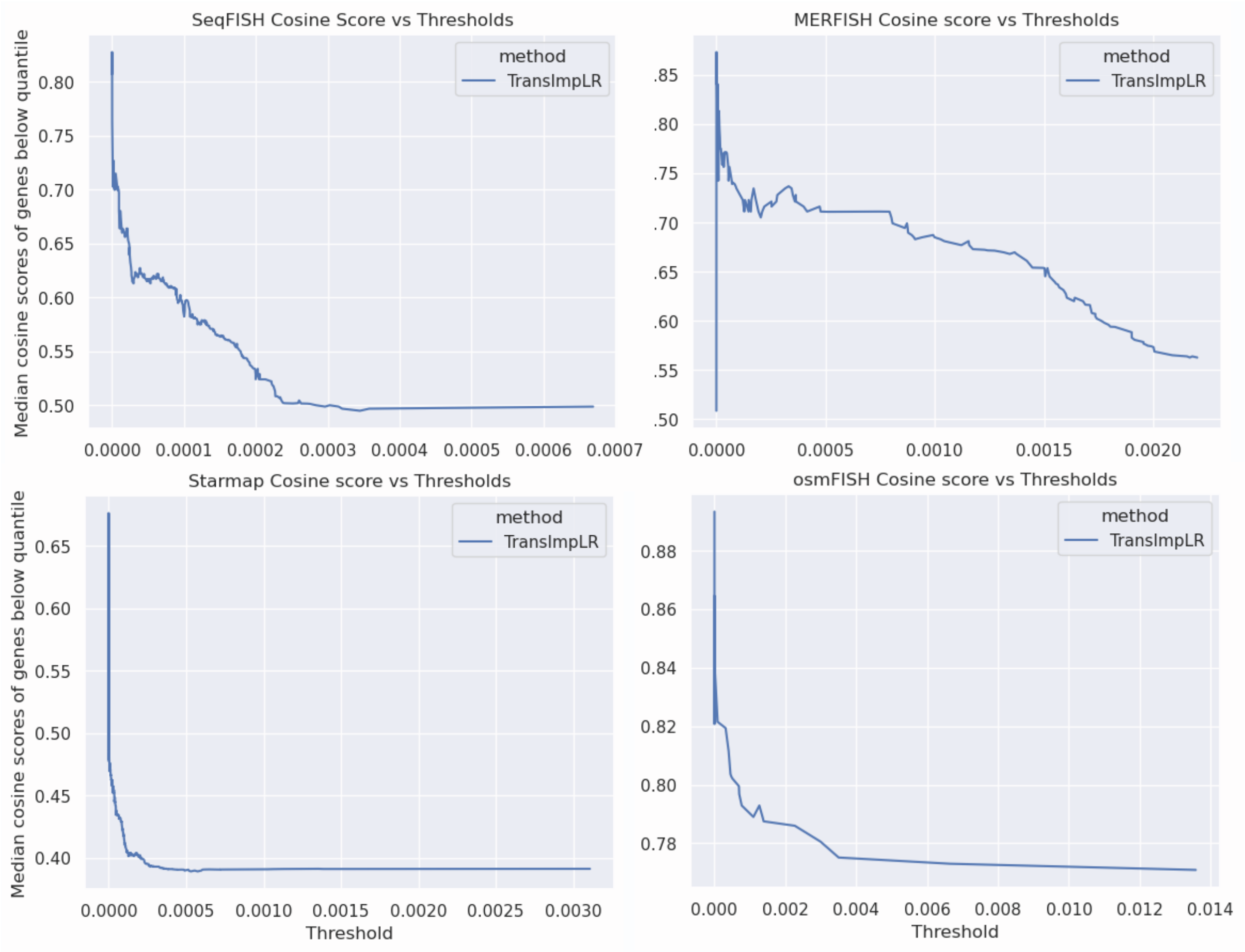

Figure S2: Cosine similarity score (y-axis) vs threshold (x-axis) of predicted variance (values are generally small after square). Similar plots can help determine a threshold for selecting genes based on the predicted uncertianty score for gene imputations.

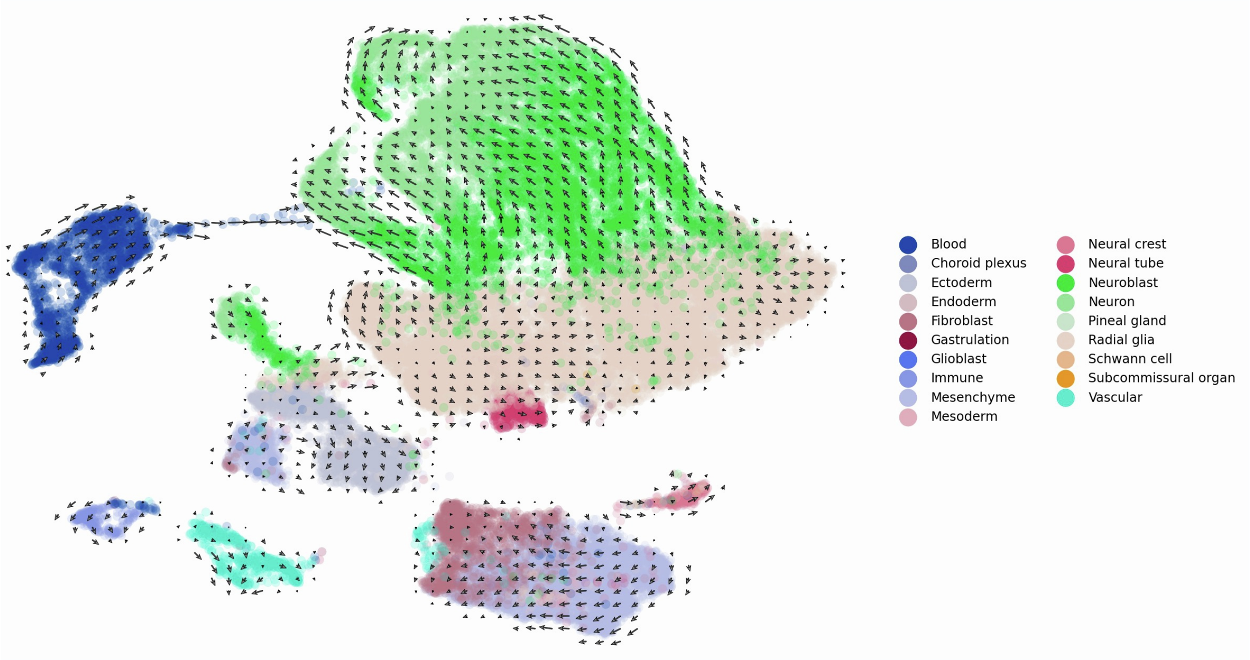

Figure S3: Cellular transition grid of the Mouse Brain data set based on RNA velocity estimated at the single cell level.

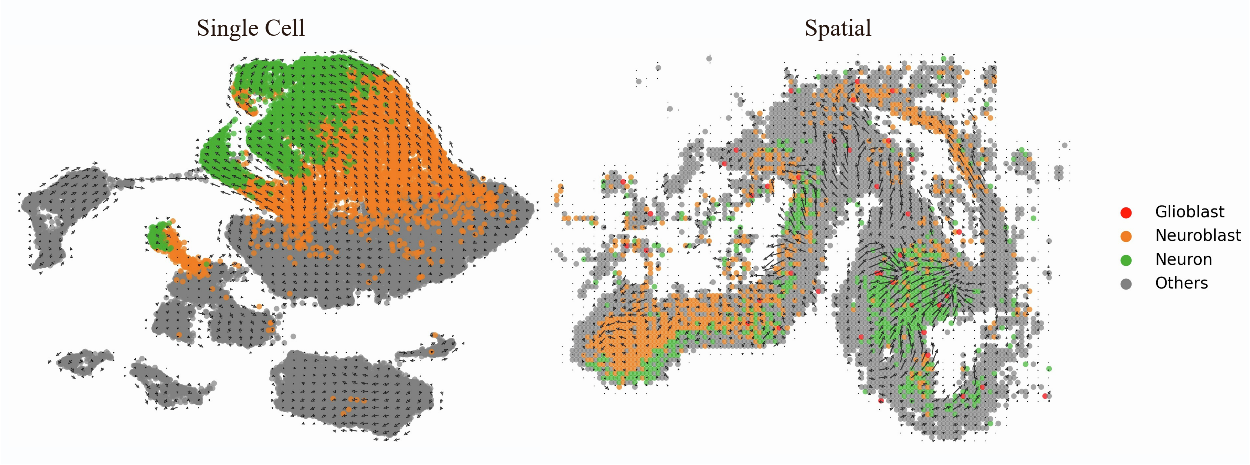

Figure S4: Cellular transition grid of the Mouse Brain at both single-cell and spatial levels colored by four cell types: Glioblast, Neuroblast, Neuron and others.

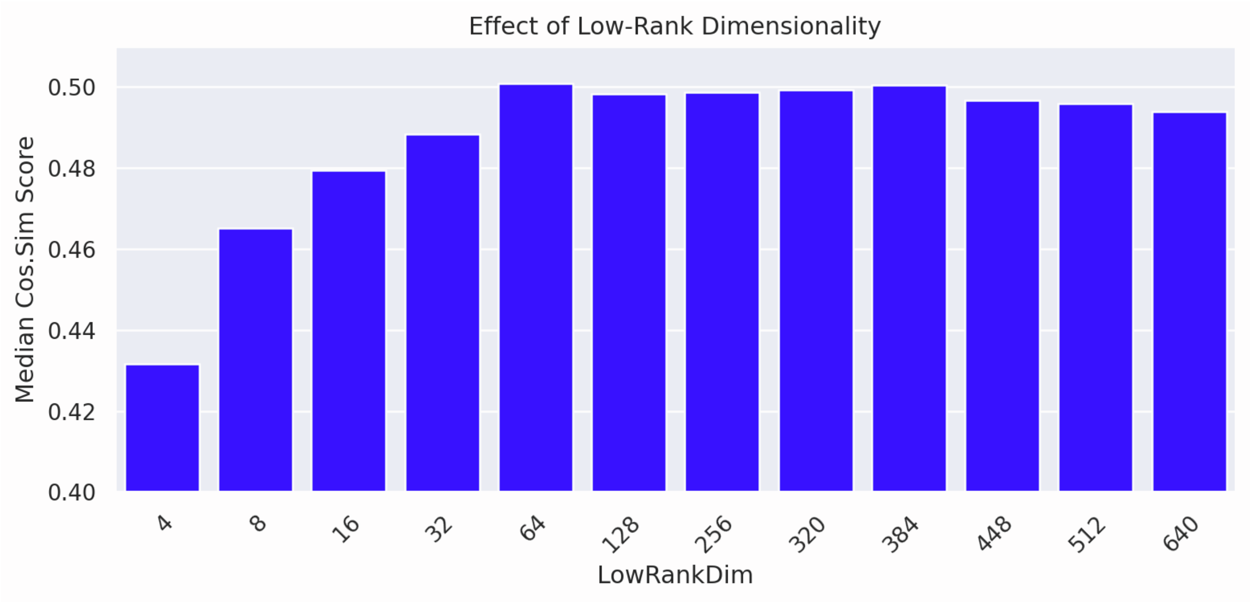

Figure S5: Effect of latent dimension on model performance.

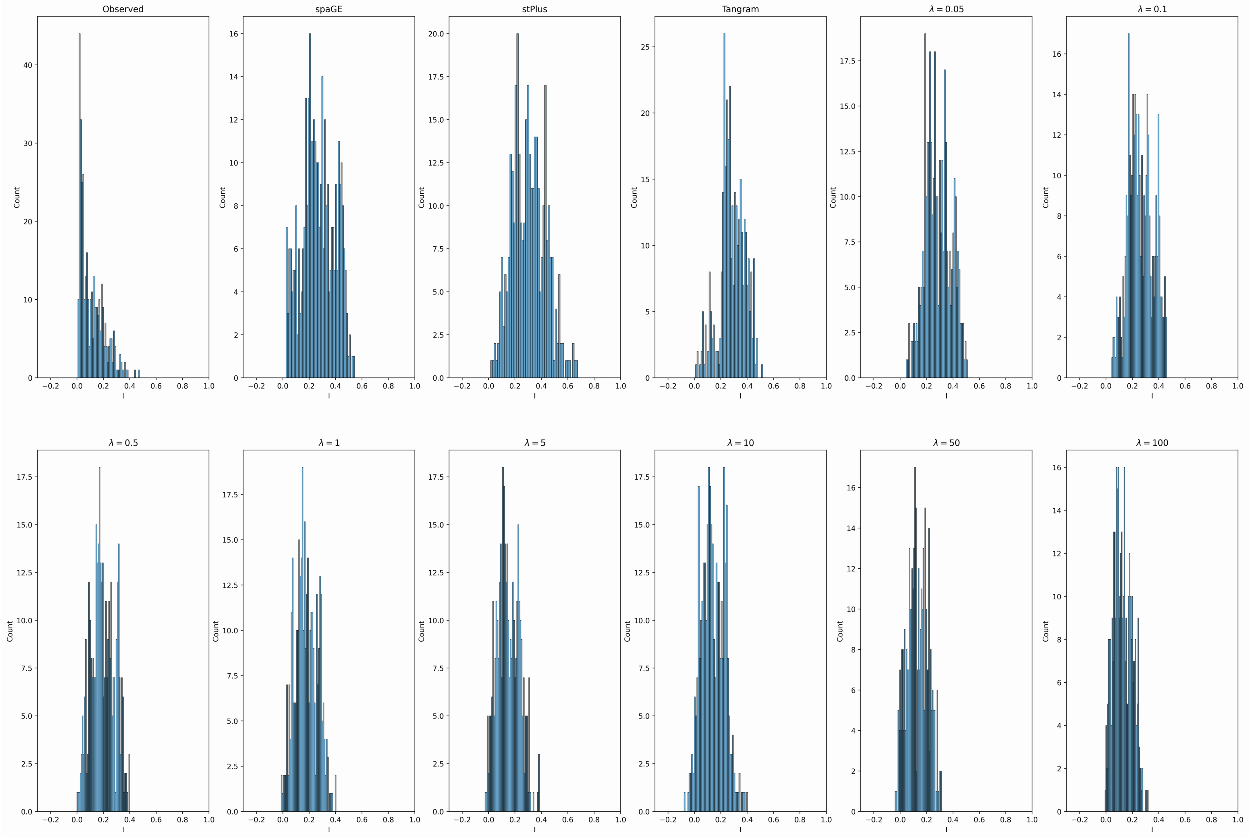

Figure S6: Distributions of Moran's Is of observed and imputed ST data. The figure shows Moran's I measures of the observed and all the imputed ST genes. While observed Moran' Is have a mode close to zero (majority genes are less spatial highly variable), spaGE, stPlus and Tangram tend to exaggerate the spatial patterns in their imputations for most genes. By gradually strengthening spatial regularization (increase lambda), TransImp pushes the modes closer to that of the observed result, thus it provides a way towards inhibiting overestimation of spatial patterns.

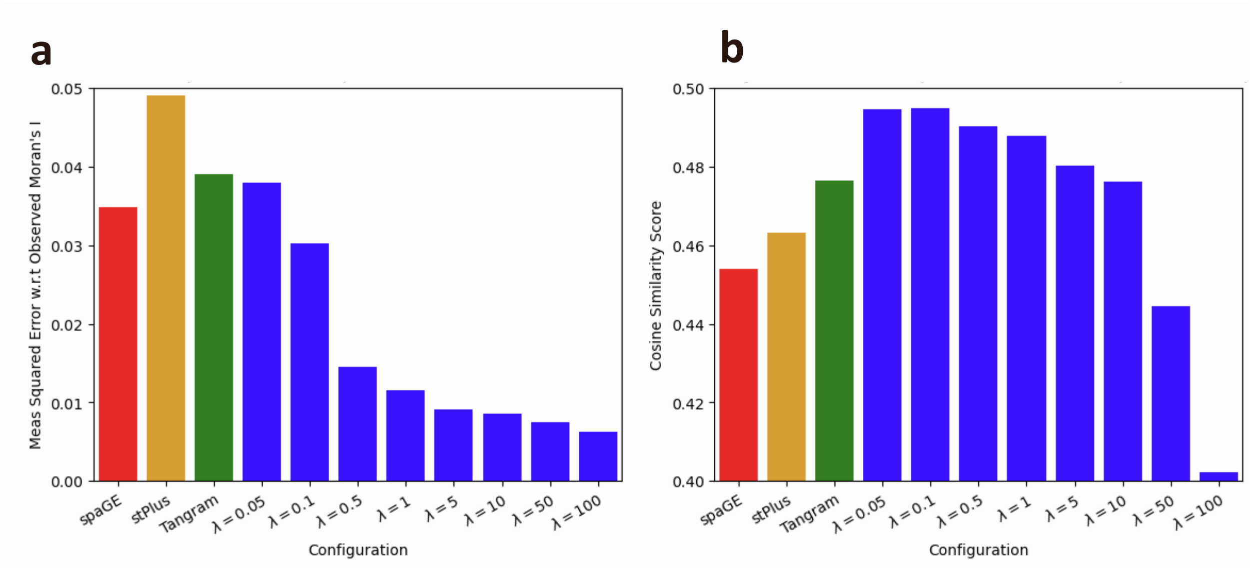

Figure S7: Effect of Spatial regularization. a. Effect of Spatial regularization on Moran's I measured in Mean Squared Error between imputed and observed ST data. b. Effect of Spatial regularization measured in Cosine Similarity Scores between between imputed and observed ST data. Fig. a demonstrates that with stronger spatial regularization, errors of spatial patterns are smaller. However, too large a spatial regularization may hurt imputation performance measured in cosine similarity, as indicated in Fig. b. A better method should have a balance with relatively low MSE of spatial patterns and high cosine similarity score.

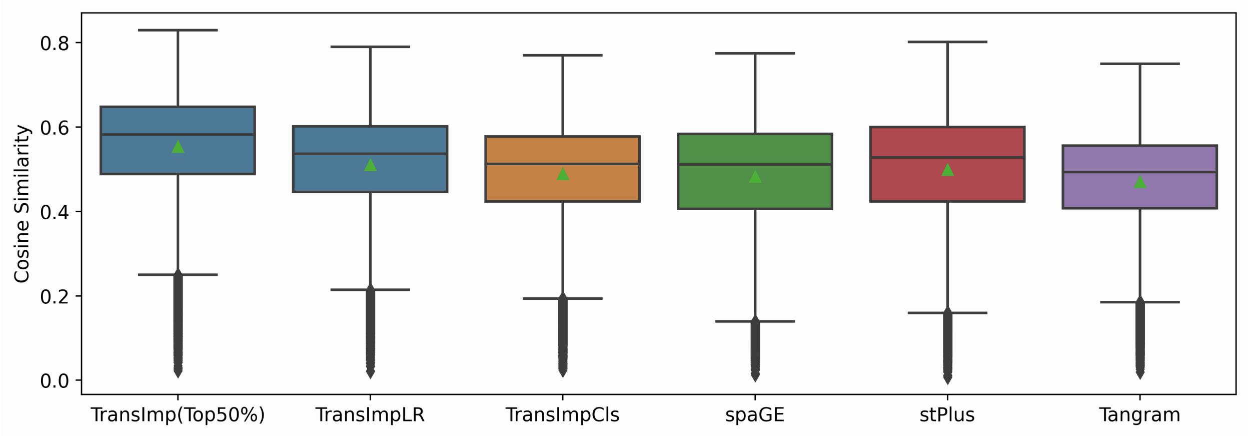

Figure S8: Cell-level median cosine similarity scores on SeqFISH benchmark.

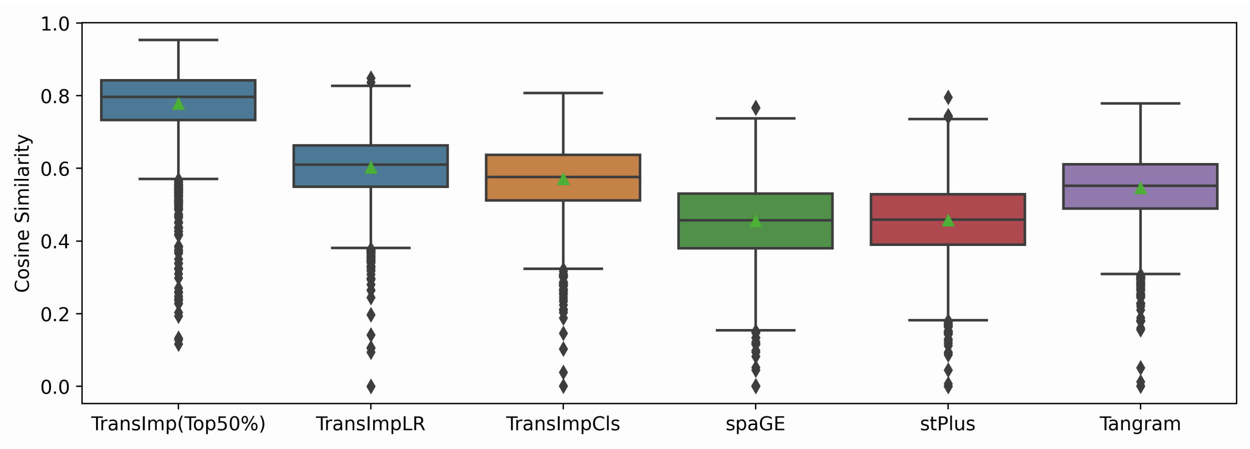

Figure S9: Cell-level median cosine similarity scores on osmFISH benchmark.

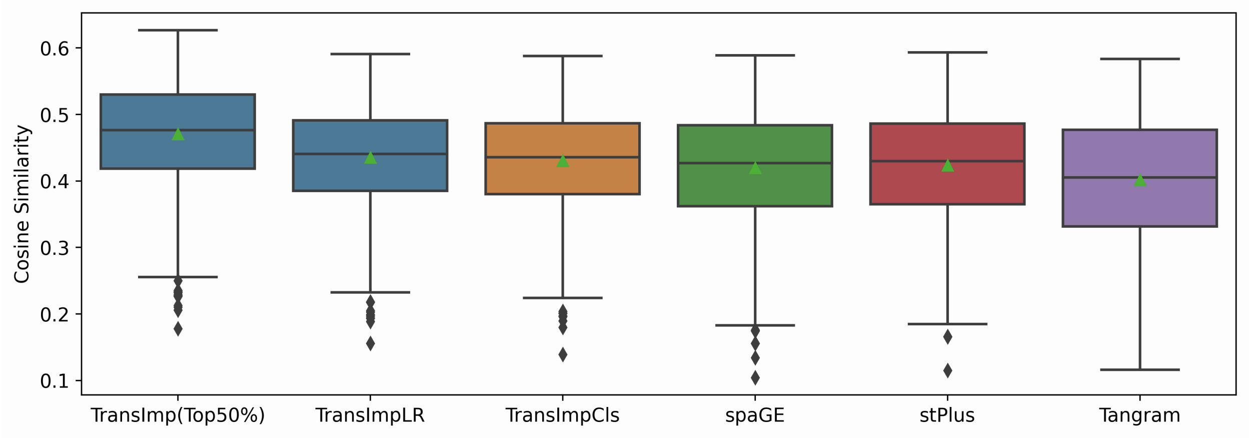

Figure S10: Cell-level median cosine similarity scores on starmap benchmark.

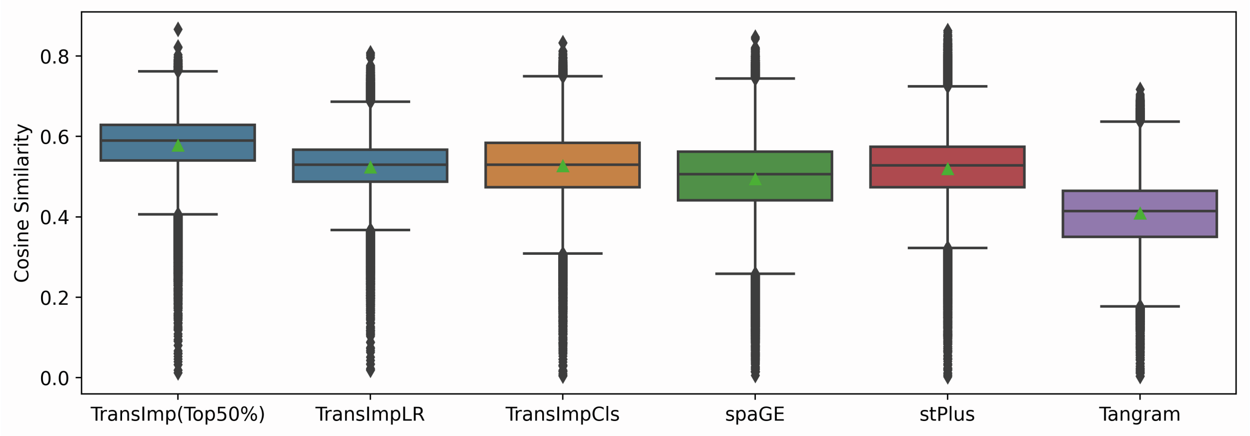

Figure S11: Cell-level median cosine similarity scores on MERFISH benchmark.

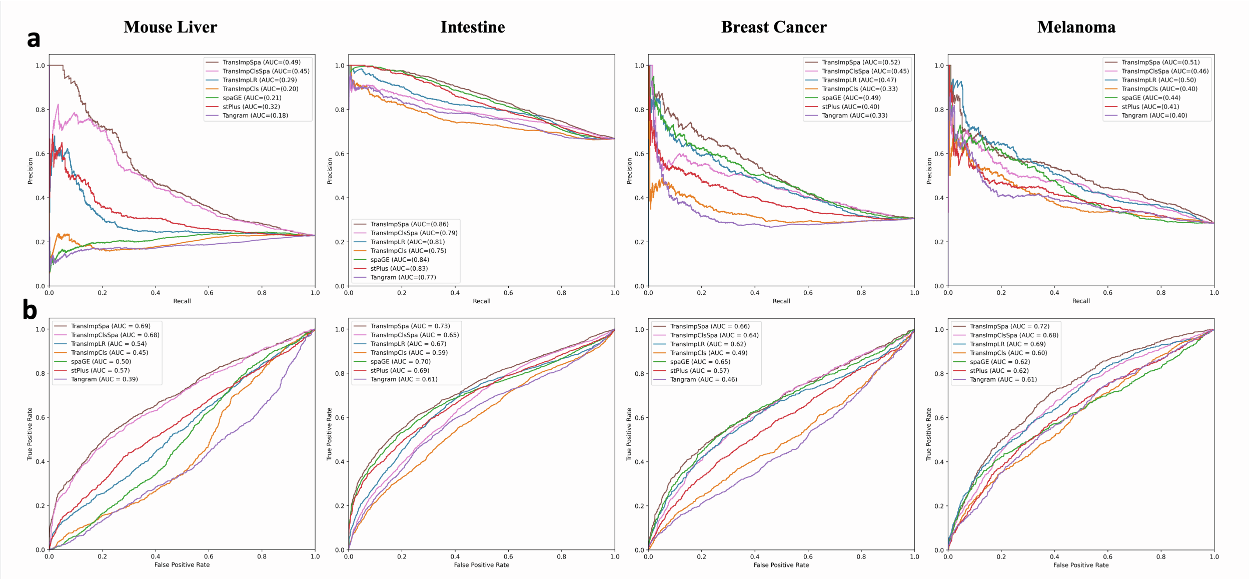

Figure S12: Results of spatially-highly variable gene detection on imputed Visium ST datasets using Moran's I test (FDR < 0.01). a. Precision-recall curves; b. Receiver Operating Characteristic curve.

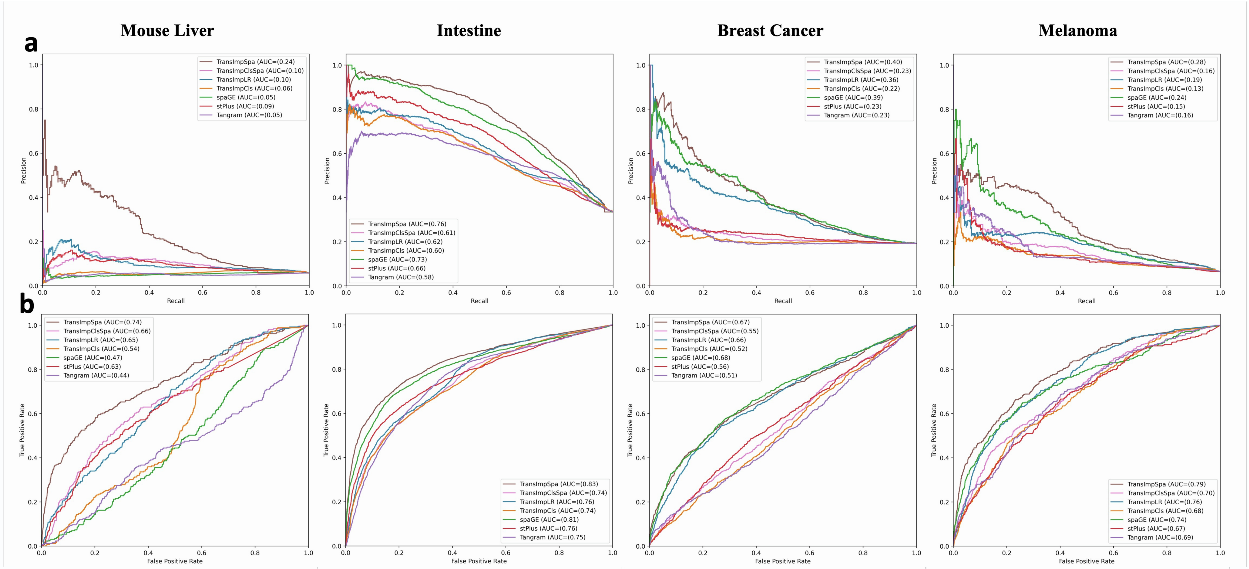

Figure S13: Results of spatially-highly variable gene detection on imputed Visium ST datasets using Spark-X test (adjusted PVal < 0.01). a. Precision-recall curves; b. Receiver Operating Characteristic curves.

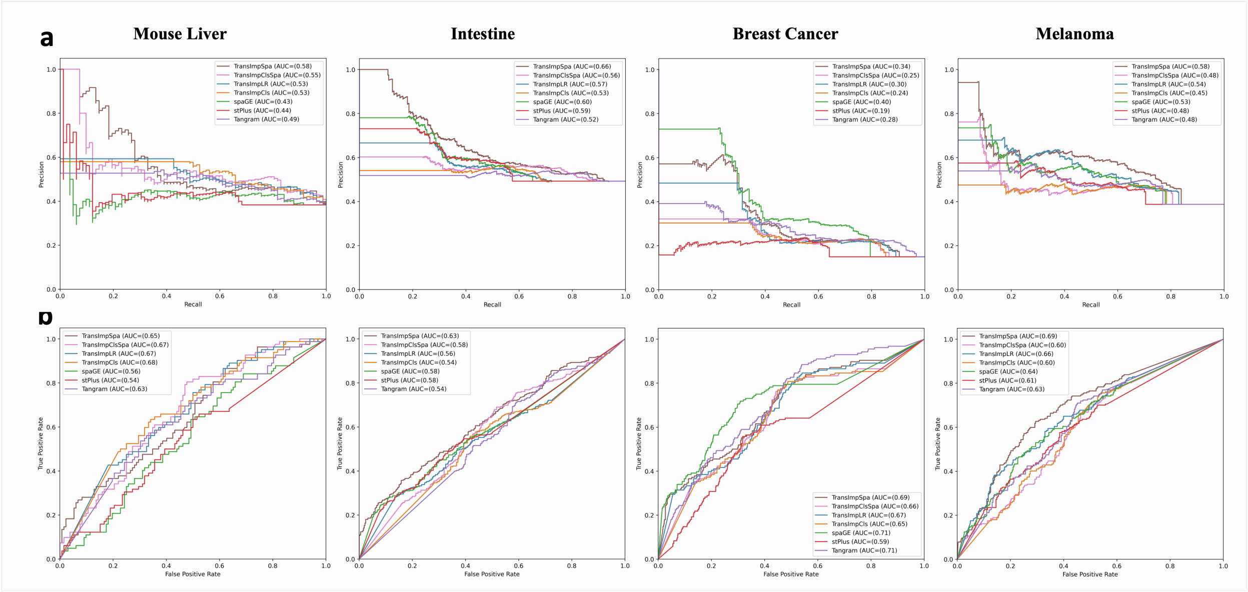

Figure S14: Results of spatial ligand-receptor pair detection on imputed Visium ST datasets using SpatialDM (FDR < 0.01). a. Precision-recall curves; b. Receiver Operating Characteristic curves.
